# Reduction/oxidation-phosphorylation control of DNA binding in the bZIP dimerization network

**DOI:** 10.1186/1471-2164-7-107

**Published:** 2006-05-04

**Authors:** Gregory D Amoutzias, Erich Bornberg-Bauer, Stephen G Oliver, David L Robertson

**Affiliations:** 1 Centre for the Analysis of Biological Complexity, Faculty of Life Sciences, The University of Manchester, Michael Smith Building, Oxford Road, Manchester, M13 9PT, UK; 2 Department of Ecology and Evolution, University of Lausanne, Lausanne, 1015, Switzerland; 3 Bioinformatics Division, Institute of Botany, School of Biological Sciences, University of Münster, Schlossplatz 4, D4814P, Germany

## Abstract

**Background:**

bZIPs are transcription factors that are found throughout the eukarya from fungi to flowering plants and mammals. They contain highly conserved basic region (BR) and leucine zipper (LZ) domains and often function as environmental sensors. Specifically, bZIPs frequently have a role in mediating the response to oxidative stress, a crucial environmental signal that needs to be transduced to the gene regulatory network.

**Results:**

Based on sequence comparisons and experimental data on a number of important bZIP transcription factors, we predict which bZIPs are under redox control and which are regulated via protein phosphorylation. By integrating genomic, phylogenetic and functional data from the literature, we then propose a link between oxidative stress and the choice of interaction partners for the bZIP proteins.

**Conclusion:**

This integration permits the bZIP dimerization network to be interpreted in functional terms, especially in the context of the role of bZIP proteins in the response to environmental stress. This analysis demonstrates the importance of abiotic factors in shaping regulatory networks.

## Background

Control of gene expression at the transcriptional level is vital and several mechanisms exist that may regulate the DNA binding of a transcription factor (TF). These include differential heterodimer formation, methylation of the DNA target site [1], phosphorylation in the TF DNA-binding domain (DBD) [2], reduction/oxidation (redox) of the DBD [3], the concentration of cations (particularly magnesium) in the nuclear environment [4]. A combination of differential heterodimer formation together with the phosphorylation and the redox mechanisms may yield complex behaviours that determine the expression or inhibition of downstream targets. We are particularly interested in the complex behaviour that these 3 mechanisms create in the bZIPs, since these TFs are involved in cell proliferation and apoptosis.

**Figure 1:**
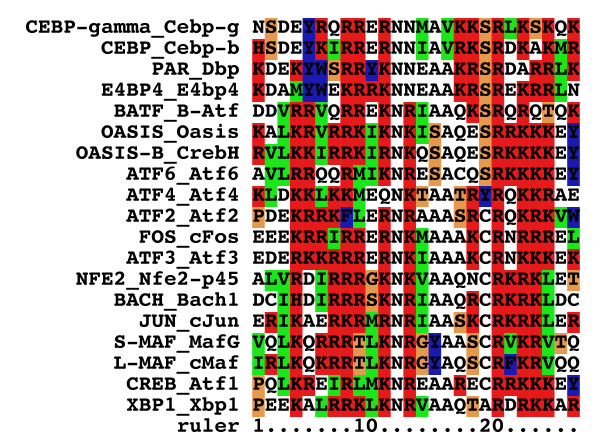
**Multiple alignment of the DNA-binding BR of human bZIPs**. Each protein sequence is a representative of a bZIP family. The first part of the protein name designates the family, while the second part designates the specific protein. The sequences are coloured by residue, using the default colouring scheme of ClustalX.

bZIPs are eukaryotic TFs, found in fungi, plants and animals. They are named for the highly conserved basic region (BR) and leucine zipper (LZ) domains, found in all these proteins. Specifically, the bZIP domain is responsible for dimerization mediated by the LZ region and DNA-binding is mediated by the BR, which is N-terminal to the LZ. Two mechanisms that control DNA binding by bZIP dimers are phosphorylation and redox. This control is mediated by specific and conserved amino acids in the BR of the protein. Serine and cysteine residues mediate phosphorylation and redox, respectively, at position 19 of the BR domain (Figure 1). We designate these residues as S19 and C19, respectively. Crystallographic analysis of various bZIP proteins, like JUN-FOS, GCN4, PAP1 and C/EBPα, has shown that the amino acids at this position make contacts with the DNA [5], [6].

**Figure 2:**
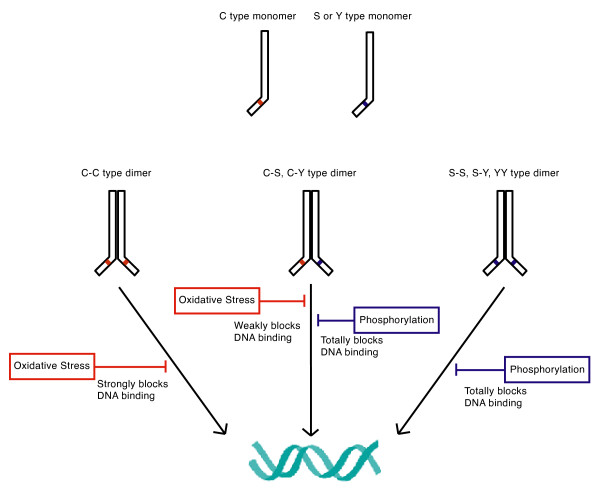
**Representation of the phosphorylation and redox control of DNA binding in bZIP dimers**. Red-dotted monomers contain C19, whereas blue-dotted monomers contain either S19 or Y19. C-C type dimers are very sensitive to oxidative stress and cannot bind to DNA under these conditions. C-S and C-Y dimers are moderately sensitive to oxidative stress. Furthermore, C-S and C-Y dimers can be phosphorylated at S19 or Y19 to totally abolish DNA binding. S-S and S-Y dimers also totally abolish DNA binding when phosphorylated at any of the S19 or Y19 residues. Phosphorylation behaves dominantly. To simplify the graph, we excluded A19 monomers and dimers. A-A type dimers are neither controlled by redox nor by phosphorylation. S-A type dimers are controlled by phosphorylation. No C-A or Y-A type dimers have been observed, see [19].

Phosphorylation of S19 in BATF [7] and C/EBP [2] proteins has been shown to add a negative charge to the positive BR and inhibit binding with the negatively charged DNA. Phosphorylation of only one of the two S19 residues in a heterodimer is sufficient to block DNA binding, thus having a dominant effect (Figure 2). It has also been postulated that the corresponding tyrosine residue (Y19) in ATF4 could potentially be phosphorylated [7].

Another mechanism of switching on or off DNA binding is the reduction/oxidation of the cysteine residue in position 19 of the BR of bZIPs (C19) [3], [8]. Oxidation of C19 blocks DNA binding, and this mechanism has been shown to operate for the AP1 proteins. Several mechanisms have been proposed or predicted for the oxidation of C19, such as reversible formation of sulphenic acid, a disulphide bond [9], S-glutathiolation [10], or S-cystenyl cystenylation [11]. Furthermore, C19 can either be protected from oxidation by the MBF1 co-activator [11], or it can be switched back from its oxidised to its reduced state by the ref-1 protein [8].

The importance of these two signalling mechanisms (redox versus phosphorylation) is stressed by the conservation of the cysteine or serine at this position. Deppmann *et al*. [7] report 55% and 35% occurrence of cysteine and serine, respectively, in position 19 in an alignment of human bZIPs. Here, we provide a more detailed phylogenetic analysis using several vertebrate and invertebrate species and highlight the level of conservation of these amino acids, strongly suggesting functional conservation. In addition to conservation at the sequence level, the same cysteine residue and the redox mechanism have been demonstrated experimentally in both human and *Drosophila *AP1 proteins [3], [11], [12]. For the phosphorylation mechanism, the importance of S19 has been shown experimentally for BATF and C/EBP proteins [2], [7], [14]. In addition, mutation of the cysteine or serine in position 19 does not affect heterodimerization properties or DNA-binding recognition [3], [7], [12]. This mutation appears to affect the selection of the signal (phosphorylation or oxidative stress) that turns on or off the DNA-binding ability of the dimer. Therefore, the conservation of the amino acids in position 19, and the report of functional conservation across different families, strongly indicates that the same mechanism is preserved from *Drosophila *to humans across many divergent evolutionary lineages.

In this work, we provide a visualization of the bZIP dimerization network and show the level of conservation of C19 and S19 residues across different phylogenetic lineages. It has yet to be established experimentally, for all bZIP proteins, whether they are under redox or phosphorylation control. However, given that all bZIP DNA-binding regions around the C19 and S19 residues have fundamentally similar properties, and extrapolating from experiments on JUN, FOS, C/EBP and BATF proteins, we predict the bZIP proteins in which the redox and phosphorylation mechanisms are utilised. Based on these predictions, we propose that the control mechanism is linked to the evolutionary history of the bZIP families. Interestingly, certain dimerization types are over-represented, while others are under-represented – suggesting strong preference for particular interaction patterns. Specifically, C19 monomers tend to dimerize with other C19 monomers, probably in order to retain redox control and rarely interact with other monomers. While dimerization with other monomer types can occur, such dimers usually have a repression function, so as to avoid inappropriate gene expression.

## Results and discussion

**Figure 3:**
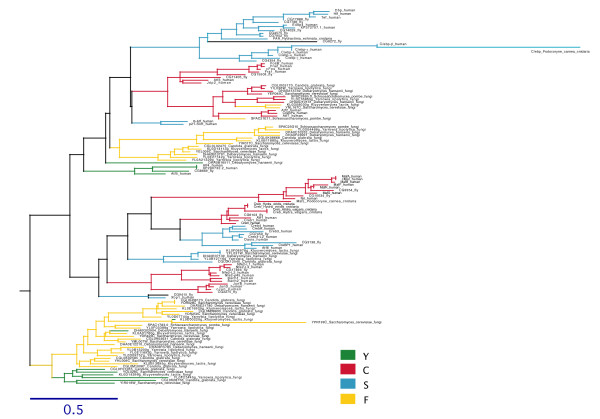
**Neighbour joining tree of the Basic region of human, fly, cnidarian and fungal bZIPs**. The colouring of the clades is based on the amino acid at position 19 (green – tyrosine; red – cysteine; blue – serine; yellow – phenylalanine).

The C19 residue of the BR alignment (Figure 1) is strikingly conserved in all vertebrates and invertebrates in the ATF2, ATF3, NFE2, BACH, CREB, FOS, JUN, L-MAF, S-MAF families (for a complete alignment of the BR domains of all bZIP proteins, see Additional file 1). S19 is also strikingly conserved in the ATF6, BATF, C/EBP, C/EBP-γ, E4BP4, OASIS, OASIS-B and PAR families. Y19, which could potentially be phosphorylated, is conserved in the ATF4 family. The XBP1 family is the only family that has a serine residue in the arthropods; this has been mutated to an alanine residue in chordates, suggesting loss of phosphorylation control in the BR. Interestingly, we see the presence of serine and cysteine residues in fungal bZIPs as well as the presence of phenylalanine (F19), which is restricted to the fungal lineage (Figure 3). This phenylalanine is necessary for recognition of the yap DNA-binding motif by the Yap proteins in fungi [15]. Nevertheless, the DNA-recognition property of this phenylalanine does not necessarily extend to the serine and cysteine residues, since they have no aromatic ring that can get stuck in the DNA. The presence of these particular serine and cysteine residues in both metazoan and fungal proteins emphasises the ancient nature of phosphorylation and redox control in bZIPs.

**Figure 4:**
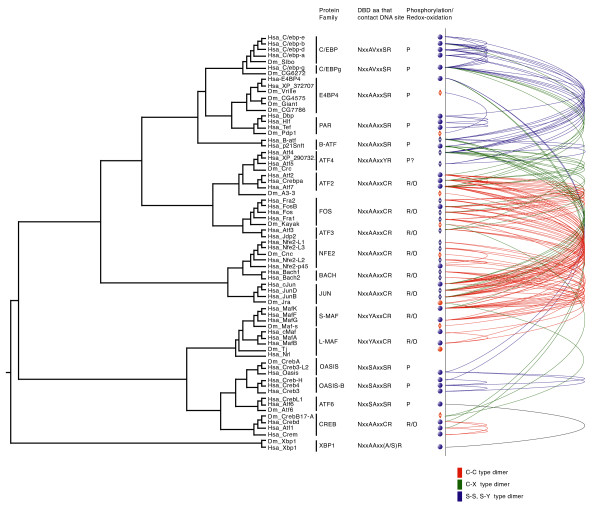
**Proportional tree of the human and fruit fly bZIP basic regions**. Additional information is added for the five amino acids in the BR that make contact with DNA [5], the presence of a phosphorylation or redox mechanism (depending on the amino acid at position 19) and the dimerizing partners of each protein. Blue circles and diamonds represent human bZIPs, and red circles and diamonds represent *Drosophila *bZIPs for which interaction data are available. Circles represent proteins that can homodimerize, while diamonds represent proteins that cannot homodimerize. The arcs to the right that connect them represent heterodimerization. The colours of the arcs represent the type of heterodimer formed (red, C-C; green, C-X; blue, S-S and S-Y).

It appears that the highly conserved nature of these amino acids is due to functional reasons (phosphorylation, redox) and not due to size restrictions at the protein-DNA interface. While cysteine, serine and alanine are all very small amino acids, the presence of tyrosine and phenylalanine in the same position shows clearly that residue size is not a major constraint. Cysteine and serine are only one point mutation apart in the genetic code and so, if size was the only constraint, we might expect these two amino acids to occur with equal frequency at this site, even within families. Thus, the conservation of the same amino acid across all the family members, from vertebrates to invertebrates (with the exception of only the XBP1 family) strongly suggests that biochemical function is involved. Phylogenetic analysis of the BR (Figure 3 &4) indicates that the use of phosphorylation or redox control has emerged several times, as the various bZIP families emerged in early metazoan evolution. Therefore, it is impossible to determine conclusively whether phosphorylation or redox control is the ancestral state at position 19.

**Figure 5:**
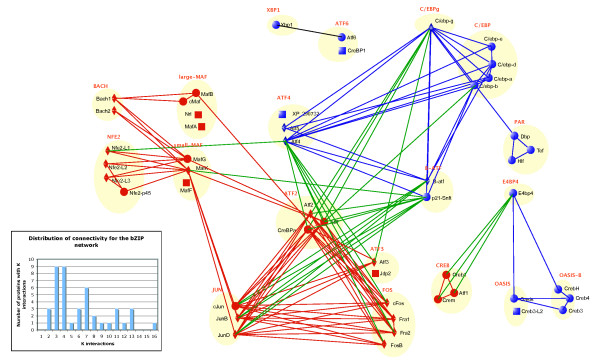
**The bZIP dimerization network, integrating phosphorylation and redox information**. Circles, diamonds and rectangles represent proteins that can homodimerize, cannot homodimerize, or for which no interaction data are available respectively. Proteins that contain C19 are coloured red, proteins that contain S19 or Y19 are coloured blue and proteins that contain A19 are coloured black. Interactions (vertices) that form C-C type dimers are coloured red, interactions that form C-X type dimers are coloured green, interactions that form S-S, S-Y or S-A type dimers are coloured blue. The bottom left diagram depicts the distribution of connectivity for the bZIP network, which does not appear to decay in a power-law.

The bZIP network is visualised in Figure 5. The bZIP dimerization network does not have the same statistical properties as all the other protein interaction networks studied to date [16]. Unlike them, it is not described by a power-law distribution (see figure 5), where the number of genes with k interactions should decay exponentially. We have previously described a network [17] of similar magnitude (the bHLH dimerization network) that followed a power-law distribution and which seems to work as a multi-switch [18]. In the case of the bHLH network, the majority of the proteins need to heterodimerize with one of the ubiquitously expressed hub proteins (eg. E2A, ARNT) in order to form functional dimers. In contrast, in the bZIP network, a large number of proteins can form homodimers. Therefore, if the bZIP network is different, what are its purposes and functions?

Integration of protein interaction data with sequence analysis reveals the different types of dimers and how many of them are formed in humans (Table 1). DNA binding of all dimers that contain at least one S19 is probably controlled by phosphorylation. The same may also happen for dimers that contain Y19, since this tyrosine can potentially be phosphorylated [7]. All the dimers that contain at least two C19 residues are probably controlled by the redox mechanism. Dimers that contain only one C19 should be deficient in redox control, according to [12].

The immediate question is whether there is a pattern to the dimerization partners that is related to the phosphorylation and redox mechanisms. To test whether the distribution of different types of dimers is significantly different from random (null hypothesis), a Chi-squared test was used (Table 2). According to the null hypothesis, the same number of proteins with the same frequency of serine and cysteine residues could dimerize randomly. Note, this statistical analysis must be treated with caution since differential gene expression and protein expression could distort the null hypothesis. By using the frequency of each amino acid in the 43 human bZIPs for which interaction data are available [19], we looked for all the types of bZIP dimers that could potentially form if all potential pairs were co-expressed. We were particularly interested in the redox control and therefore focused on the presence of C19-bearing dimers. We observed biases, particularly an over-representation of C-C type dimers that also resulted in an under-representation of C-X dimers (dimers that contain only one C19) and *vice versa *(Table 2).

It is conceivable that this under-representation of C-X dimers is an artefact and this could have arisen in two different ways. Firstly, we explored the possibility that C19-bearing proteins tend to homodimerize more than the others. Secondly, we explored the possibility that C19-bearing proteins tend to dimerize more with their closest homologues, that is proteins of the same family that also have a cysteine at position 19. In order to exclude biases created by these two options, we also performed the chi-squared test for heterodimers only, and for heterodimers that are not close paralogues (Table 2). In both cases, the over-representation of C-C and under-representation of C-X dimers is statistically significant.

In the above statistical tests, we used all the paralogues of each family. Nevertheless, most paralogues have similar dimerization patterns. If position 19 is not actually linked to the dimerization pattern, but is only responsible for family-specific DNA core-site recognition, then gene duplication (within a protein family) could have caused an artefactual connection. In order to exclude this possibility, all the paralogues of each family were collapsed into one such that the network was then composed of interacting families. We retained the structure of the network among the families, but we shuffled the amino acids of position 19 across the various families 10,000 times. This model showed clearly that, in less than 2% of the shuffled networks, did we obtain an under-representation of C-X family dimers similar to that observed in the data (11 or fewer families forming C-X dimer types) (see also Additional file 2). Furthermore, we performed the same analysis in other positions of the BR alignment (positions 9, 13, 15, 16, 23) that are also strongly conserved within each family, but we did not observe any under/over-representation of amino acid combinations at a cut-off level of 5% (see also Additional file 2), in contrast to what we observed for position 19. It should also be stressed that the experimental evidence from [20] shows that mutation of C19 did not affect DNA binding, DNA element recognition, or dimerization of the Zta bZIP protein.

Interestingly, it is apparent that the C-X heterodimer type is not favoured. This can be explained by the dominant nature of phosphorylation over redox control. The presence of only one S19 residue would be sufficient to place the DNA-binding properties of the dimer under phosphorylation control. The loss or decrease of the redox mechanism in the BR is known to increase the transforming activity of the JUN-FOS heterodimer [12], [13]. Thus, it is presumably important to retain the redox mechanism and avoid heterodimerizing with other types (see Figures 4 &5).

What is the function of the C-type bZIPs that actually dimerize with other types (X-types), thus forming C-X type dimers and exhibiting insufficient redox control? By examining the activation/inhibition activity of the X-type partners in general, it appears that E4BP4 [21], p21-SNTF [22] and BATF [7] have an inhibitory effect when dimerizing with other factors. In the case of E4BP4, this is due to the active repression domain that it possesses. The cases of ATF4, C/EBP-β and C/EBP-γ are more complex because they can exhibit activating or inhibiting effects, depending on post-translational modification [23], alternative splicing [24], or the cell type in which they are expressed [25]. Nevertheless, they do have the ability to function as inhibitors. It is reasonable to assume that, for C-type molecules, it is generally acceptable to escape from the redox control, as long as they dimerize with an inhibitor, or if the new heterodimer cannot recognise and bind to promoters of downstream targets that need to be controlled by the redox mechanism – thus preventing uncontrolled activation of downstream targets.

Dimerization is an important mechanism for generating complex behaviour with a limited number of protein "building blocks". Work on other dimerizing TF families, like the bHLH, has revealed a dimerization network with a hub-based structure [17] that seems to work as a multi-switch [18], especially in development and the cell cycle. A very different network structure was found for the bZIP proteins, despite the fact that they share a similar crystal structure with the bHLH proteins. Interestingly, there seems to be a pattern in the formation of dimers in the bZIPs (Figures 4 &5). These results indicate that environmental signals (and, particularly, oxidative stress) could have imposed some selective pressure on the dimerizing properties of these proteins. Alternatively, the dimerizing properties of each monomer could have imposed some pressure on the presence of cysteine or serine in position 19 of the BR. The redox mechanism has been implicated in the regulation of DNA binding in other TF proteins: p53, Sp1, NFI, NF-κB, PEBP2/CBF, the nuclear receptor proteins (oestrogen and glucocorticoid receptors) and the bHLH protein, USF (reviewed in [26]). When cells undergo oxidative stress, the cell cycle is affected and it seems that the redox control of cysteine residues in the DNA-binding domain of various TFs is a simple (but very efficient) mechanism of transducing environmental signals to the transcriptional machinery. In addition, oxidative stress has been implicated in the aetiology of several human diseases, like cancer, ischemia, atherosclerosis, neurodegenerative disorders and ageing (reviewed in [27]). It will be of great interest to further enhance our understanding of how this mechanism works and affects other dimerizing TF families, like the bHLH and nuclear receptors, determining whether this pattern is global or restricted to the bZIPs.

## Conclusion

The integration of genomic, phylogenetic and functional data reveals a preference in the interaction partners of bZIP proteins that is linked to oxidative stress. Specifically, bZIP proteins whose DNA binding is controlled by redox tend to dimerize, with a frequency more than that expected by chance, with other bZIP proteins that are also controlled by redox. These results demonstrate that abiotic factors may play a major role in shaping regulatory networks. While the dimerization networks of bHLH proteins and nuclear receptors are hub-based, that of the bZIP proteins is not. Nevertheless we have demonstrated that this network is not random. It follows a logic which strongly links its structure with the network's functional role in environmental sensing.

## Methods

Protein-protein interaction data for all the human bZIPs were taken from [19]. In that study, the coiled-coils of 43 out of the 51 human bZIPs that we had identified were checked for the presence of an interaction with any of the other bZIP coiled-coils. Each protein was used both as a surface-bait and a probe. Therefore, a given heterodimerization is represented by two different symmetrical (across the diagonal) points in an interaction matrix. We considered an interaction as valid if its Z-score was greater than 2.5 in both directions, where the Z-score is a measure of the signal-to-noise ratio [19].

bZIP sequences were obtained by genome-wide scanning using custom-made HMMs [28]. The training of the HMM models was based on protein sequences annotated as bZIPs in the TRANSFAC database (version 4) [29]. Four vertebrate (*Homo sapiens*, *Gallus gallus, Takifugu rubripes, Danio rerio*), four invertebrate (*Ciona intestinalis*, *Drosophila melanogaster, Apis melifera, Anopheles gambiae*) and six fungal (*Schizosaccharomyces pombe, Yarrowia lipolytica, Debaryomyces hansenii, Kluyveromyces lactis, Candida glabrata, Saccharomyces cerevisiae*) genomes were scanned for bZIP sequences. In addition, cnidarian bZIP sequences were retrieved, using a keyword search, from the NCBI protein database. The inclusion of a sequence as a bZIP 'hit' required both the presence of an LZ and a typical DNA-binding region, as defined by [30] and the 2ZIP program [31]. This strict criterion was imposed because LZ domains may appear by chance, due to abundance of the leucine residue and the short length of the domain [32].

Multiple sequence alignments were performed for family members with T-COFFEE [33] (Notredame *et al*., 2000) and among different families using CLUSTALW [34] (Thompson *et al*., 1994). The alignment was based on the BR and LZ domain. Phylogenetic analysis (neighbour-joining) of the BR was performed by the PHYLIP package [35], using the PROTDIST and NEIGHBOUR programs, using the JTT model of amino acid replacement. The neighbour-joining tree was visualised with TreeEdit [36].

### Classification of bZIPs in protein families

We classified the 51 human bZIPs into 19 families, based on the neighbour-joining phylogenetic analysis of the BR-LZ domain, combined with the distribution of orthologues and the domain architecture of the whole sequence (G. D. Amoutzias, PhD Thesis, The University of Manchester, 2005). Specifically, the designations of our analysis were based on the presence of invertebrate orthologues and distinct domain architectures for each family. The new designations are: (1) the split of the OASIS family into OASIS and OASIS-B, (2) the split of CNC family into NFE2 and BACH, and (3) the split of the C/EBP family into C/EBP and C/EBP-γ.

## Authors' contributions

GDA carried out all of the bioinformatics analyses and wrote the manuscript, together with SGO. EBB, SGO and DLR conceived of the study, participated in its design and coordination. EBB and DLR revised and refined the manuscript, which all authors read and approved before submission.

## Supplementary Material

Additional File 1 Multiple alignment in phylip format. Click here for file

Additional File 2 This file contains the simulations of shuffling several conserved positions (9, 13, 15, 16, 19, 23) in the Basic Region of bZIP proteins, 10,000 times, while retaining the structure of the bZIP dimerization network. Click here for file

## Figures and Tables

**Table 1:** Types of dimers formed by the homo/heterodimerization of the 43 human bZIPs.

**Amino acid composition of dimer in position 19**	**Dominant mechanism that blocks DNA binding of the dimer**	**Number of human bZIP dimers**
A-A	Uknown	1
A-S	Phosphorylation	1
C-C	Redox	75
C-S	Phosphorylation	17
C-Y	Phosphorylation	6
S-S	Phosphorylation	37
S-Y	Phosphorylation	10

**Table 2:** Observed and expected C-X and C-C type dimers.

	**homodimers, intra- and inter-family heterodimers**			**intra- and inter-family heterodimers**			**inter-family heterodimers**		
**Dimer Type**	**E**	**O**	χ^2^	**E**	**O**	χ^2^	**E**	**O**	^2^

C-X	72.51	23	3 e^-16^	60.18	23	2 e-^11^	51.79	23	2 e^-8^
Other	74.49	124		61.82	99		53.21	82	
C-C	45.79	75	6 e^-8^	38.01	65	1 e-^7^	32.71	57	3 e^-7^
Other	101.2	68		83.99	57		72.29	48	

Abbreviations: E = expected, O = observed, χ^2 ^= Chi squared P value. C-X refers to any heterodimer that contains only one Cys 19.
